# A Postbiotic Containing *Limosilactobacillus fermentum* CNCM I-2998 and *Lactobacillus delbrueckii* subsp. *lactis* CNCM I-4831 Reduces Bowel Movement Frequency in Healthy Adults with Self-Reported Unusually Frequent and Soft Stools

**DOI:** 10.3390/microorganisms14091939

**Published:** 2026-09-02

**Authors:** Nize Otaru, Erik Eckhardt, Matthew R. Hayward, Tomoya Isaji, Yuki Kamioka, Britt Blokker, Gabriel Vinderola

**Affiliations:** 1 Health, Nutrition & Care (HNC), dsm-firmenich, 4303 Kaiseraugst, Switzerland; 2 dsm-firmenich Houdan SAS, 78550 Houdan, France; 3 dsm-firmenich, Lexington, MA 02421, USA; 4 NOF CORPORATION, Kawasaki 210-0865, Kanagawa, Japan; 5 Instituto de Lactología Industrial (Consejo Nacional de Investigaciones Científicas y Técnicas—Universidad Nacional del Litoral), Santa Fe S3000, Argentina

**Keywords:** postbiotic, *Lactobacillus*, gastrointestinal symptoms, bowel movement frequency

## Abstract

Unusually frequent and soft stools may negatively impact the quality of life. A postbiotic containing heat inactivated *Limosilactobacillus fermentum* CNCM I-2998 and *Lactobacillus delbrueckii* subsp. *lactis* CNCM I-4831 has previously been shown to alleviate gastrointestinal symptoms and diarrhea in patients with clinically diagnosed gastrointestinal disorders. This study evaluated whether this postbiotic can support bowel movement frequency in Japanese healthy adults with self-reported frequent and soft stools. In a randomized, double-blind, placebo-controlled clinical trial, participants (n = 100) received the postbiotic (20 billion heat-inactivated cells per day) or placebo for four weeks. The primary endpoint was weekly bowel movement frequency. Secondary endpoints included days with bowel movements per week, sense of relief, stool characteristics (shape, color, odor, water content), gastrointestinal symptoms, and fecal microbiome composition. After four weeks, the postbiotic group showed significantly lower bowel movement frequency and fewer days with bowel movements when compared with the placebo group. Importantly, these reductions were not accompanied by decreased stool volume or increased hard stools or constipation symptoms. Mild changes in microbiome composition were detected across groups, and no adverse events were reported. These findings suggest that the postbiotic is safe and can help reduce bowel movement frequency in healthy individuals experiencing unusually frequent and soft stools.

## 1. Introduction

Chronic diarrhea is defined as loose or watery stools which occur three or more times within 24 h and last for four or more weeks [1], corresponding to ≥21 bowel movements per week. Certain conditions such as food intolerances, non-celiac wheat sensitivity, bile acid malabsorption or irritable bowel syndrome (IBS) can cause an increase in the frequency of unusually soft stools that do not meet diagnostic criteria for diarrhea yet may negatively impact individuals though self-perceived reduction in quality of life. Additionally, an unusually high bowel movement frequency that does not qualify as diarrhea has been associated with changes in the gut microbiome [2].

The gut microbiome plays a critical role in health and disease. Growing awareness amongst the public has made microbiome modulation a thriving industry with probiotics, live microorganisms typically orally consumed, becoming one of the most popular interventions. In addition to live microbes, inanimate microorganisms have also been explored for their potential health benefits. However, validating these claims remains challenging without a standardized definition comparable to probiotics. A range of definitions has been used such as heat-inactivated probiotics, inactivated probiotics, heat-killed probiotics, tyndallized probiotics, ghost probiotics, non-viable immunobiotics, paraprobiotics, parabiotics, cell fragments, cell lysates and postbiotics [3]. In an effort to merge all these related definitions into a single one, the International Scientific Association for Probiotics and Prebiotics proposed that the term postbiotic be used to refer to a “preparation of inanimate microorganisms and/or their components that confers a health benefit on the host” [4]. The core of this definition is to refer to preparations of inanimate microorganisms, as whole, fragmented or lysed cells. Metabolites can be present or not in the preparation (as cells may have been extensively washed before inactivation). However, purified isolated metabolites do not fall under the category of postbiotics.

One notable example of a postbiotic is the mixture containing heat-inactivated *Limosilactobacillus fermentum* CNCM I-2998 and *Lactobacillus delbrueckii* subsp. *lactis* CNCM I-4831 cultures which has historically been referred to as *Lactobacillus* LB and *Lactobacillus acidophilus* LB. This postbiotic has demonstrated efficacy in managing acute and chronic diarrhea in both children and adults, and in reducing abdominal pain in adults [5,6,7]. Ex vivo stool fermentation assays have shown that *Lactobacillus* LB may promote the growth of *Bifidobacteria* in a complex community [8,9], suggesting a potential role for *Lactobacillus* LB in modulating the gut microbiome, supporting the management of gastrointestinal conditions beyond the ones already demonstrated.

The aim of this study was to test if daily administration of the postbiotics obtained with *Limosilactobacillus fermentum* CNCM I-2998 and *Lactobacillus delbrueckii* subsp. *lactis* CNCM I-4831 can reduce bowel movement frequency in a healthy Japanese cohort with self-reported unusually frequent and soft stools.

## 2. Materials and Methods

### 2.1. Investigational Product

The investigational product, *Lactobacillus* LB (dsm-firmenich, Houdan, France) was produced through co-fermentation of *Limosilactobacillus fermentum* CNCM I-2998 (formerly known as *Lactobacillus fermentum* CNCM I-2998) and *Lactobacillus delbrueckii* subsp. *lactis* CNCM I-4831. The complete fermented mixture was subjected to heat treatment to inactivate the microbial cells and dried to produce a powder (commercially available as Humiome^®^ Post LB). Each capsule contained 170 mg of powder resulting in a dose of 10 billion inactivated bacterial cell bodies. A weight matched placebo containing maltodextrin (Matsutani Chemical Industry Co., Ltd., Itami City, Japan) and crystalline cellulose (Asahi Kasei Corporation, Tokyo, Japan) was used. Placebo capsules were indistinguishable in appearance from those containing the postbiotic to maintain blinding.

### 2.2. Study Participants

This study enrolled healthy Japanese male and female adult participants with unusually high weekly bowel movements at baseline (defined as ≥10 and ≤20 weekly bowel movements) including ≥50% of stools rating on the Bristol stool scale as a type 5 (“soft blobs with clear-cut edges”) or 6 (“fluffy pieces with ragged edges”) [10].

Exclusion criteria were ongoing medical treatments or history of major and/or chronic conditions (including malignant tumor, heart failure, myocardial infarction, arrhythmia, hepatopathy, nephropathy, cerebrovascular disorder, rheumatism, diabetes, dyslipidemia, or hypertension), any diseases that may substantially affect bowel movements, implanted pacemakers/defibrillators, use of pharmaceuticals (including traditional herbal medicines) or gastrointestinal-active foods or supplements (including Foods for Specified Health Uses (FOSHU), Foods with Functional Claims (FFC), probiotics/fermented or fiber-enriched foods), intolerances or allergies to the test product, excessive alcohol intake (>20 g/day), diarrhea in the last two weeks, diagnosed IBS according to Rome IV criteria, pregnancy/lactation, enrollment in other clinical trials within the last 28 days, or investigator/physician-judged ineligibility.

### 2.3. Study Design

This study was conducted in accordance with the Declaration of Helsinki, approved by the Seishinkai Medical Corporation Takara Clinic Ethics Committee (Approved Number: 2306-00218-0106-12-TC), and registered with UMIN-CTR (registration number UMIN000051486).

A placebo-controlled, parallel group, double-blind intervention trial was conducted over four weeks, during which participants consumed two capsules daily of either placebo or postbiotic. This regimen resulted in a daily intake of 20 billion inactivated cell bodies for the postbiotic group. The primary endpoint for efficacy was the bowel movement frequency after 4 weeks of intervention. Secondary endpoints included the bowel movement frequency at 1, 2 and 3 weeks of intervention, days with bowel movements per week across the 4 weeks of intervention, stool shape, stool color, stool odor, stool water content, sense of relief, gastrointestinal symptoms, and fecal microbiome composition after 4 weeks of intervention.

The study was conducted at the Takara Clinic Seishinkai Medical Corporation (Tokyo, Japan) and included two on-site visits: a baseline visit (visit 1) and an end-of-study visit (visit 2). Between visits, participants completed daily and weekly questionnaires.

Prior to the baseline visit, informed consent was obtained and participants were provided with a stool sample collection kit for completion at home. Additionally, participants received a bowel movement diary to be completed daily and food records to be completed during the 3 days prior to the baseline visit. At baseline (visit 1), eligibility was confirmed. Assessments included peripheral blood sampling, urinalysis, a physical exam (including body mass index and blood pressure) and a medical interview. The completed bowel movement diary and food records were reviewed. Participants completed digestive health questionnaires (Gastrointestinal Symptoms Rating Scale (GSRS) and Izumo scale) and provided fecal samples for assessment of water content and microbiome profile. They were then given a new bowel movement diary, food records (to be completed during the 3 days prior to the final visit) and a stool collection kit.

Eligible participants were randomized in a 1:1 allocation ratio to one of the study groups using a stratified block random allocation algorithm in SPSS (v23), with sex as the stratification factor. The randomized participants received a 4-week postbiotic or placebo supply and were instructed to take one capsule twice daily (morning and evening, for a total of two capsules a day) with water, without chewing.

The end of study visit (visit 2) mirrored the procedure conducted at the baseline visit (visit 1), except that eligibility confirmation procedures or new bowel movement diaries, food records, or stool collection kits were not provided.

### 2.4. Assessments

#### 2.4.1. Bowel Movement Diary

All bowel movements were recorded daily by participants using a standardized bowel-movement diary. For each bowel movement, participants documented the occurrence and assessed sense of relief, stool shape, stool color, and stool odor.

Sense of relief was evaluated using a three-category scale: “feels refreshed”, “normal”, “feels like there’s still stool left”.

Stool shape was assessed using the Bristol Stool Scale which classifies stools into seven different types.

Stool color was assessed using a predefined six-category scale: “yellow”, “yellowish-brown”, “brown”, “tea-brown”, “red-brown”, and “dark brown”.

Stool odor was assessed using a five-category scale: “the smell has become quite strong,” “the smell has become slightly stronger”, “no change from usual”, “the smell has become slightly weaker”, “the smell has become quite weaker”.

#### 2.4.2. Digestive Health Questionnaires

Participants completed the GSRS [11] and the Izumo scale [12] at baseline and end of the study. The GSRS is a 15-item questionnaire including a seven-point Likert scale for each item assessing the severity of gastrointestinal symptoms experienced during the previous week. Similarly, the Izumo scale is a 15-item questionnaire including a six-point Likert scale.

#### 2.4.3. Fecal Analyses

Daily fecal volumes were estimated by using a No.7 film canister (Konchu Bunken Ropponashi, Chiyoda City, Tokyo, Japan) as a reference and recording the volume.

Fecal dry weight was assessed by weighing a fecal sample, obtained within 3 days of baseline and end of study visit, before and after complete drying in an oven at 80 °C for 48 h.

To assess the fecal microbiome profile, participants collected a fecal sample using the DNA OMNIgene^®^∙GUT OM-200 Collection Kit (DNA Genotek Inc., Ottawa, ON, Canada), which was stored at −20 °C prior to shipment for 16S rRNA gene sequencing.

DNA for microbiome analysis was extracted by mechanical lysis of cells using glass beads and subsequent purification using the GENE PREP STAR PI 1200A system (Kurabo Industries Ltd., Osaka, Japan). The V1-V2 16S rRNA gene region was amplified using the primers 16S-27Fmod (5′-TCGTCGGCAGCGTCAGATGTGTATAAGAGACAGAGRGTTTGATYMTGGCTCAG 3′) and 16S-338R (5′-GTCTCGTGGGCTCGGAGATGTGTATAAGAGACAGTGCTGCCTCCCGTAGGAGT 3′). Sequencing was performed on an Illumina MiSeq platform using paired-end chemistry with 2 × 250 bp or 2 × 300 bp.

Raw Illumina sequencing data was processed and analyzed using QIIME2 (v 2020.8) and the SILVA reference base (v138).

#### 2.4.4. Safety Laboratory Panels

Blood samples were collected at baseline and end of study visit, using the Neo Tube NP SP1016, Neo Tube NP EK0205 and Neo Tube NP FN0205 tubes (Nipro Corporation, Osaka, Japan). Safety laboratory assessments included a hematology (including white blood cell count, red blood cell count, hemoglobin, hematocrit, and platelet count) and chemistry panel (including aspartate aminotransferase, alanine aminotransferase, γ-glutamyltranspeptidase, total bilirubin, total protein, urea nitrogen, creatinine, uric acid, sodium, potassium, chloride, serum amylase, total cholesterol, high-density lipoprotein cholesterol, low-density lipoprotein cholesterol, triglycerides, glucose, and hemoglobin A1c).

Urine samples were collected at baseline and end of study visit using Urine Spitz MP 10U tubes (Toyama Daioh Sangyo Co., Ltd., Tokyo, Japan). Safety laboratory assessments consisted of standard urinalysis including urinary protein, glucose, pH levels, and occult blood.

### 2.5. Sample Size Calculation and Statistical Analyses

Minimum group sizes were estimated based on previously published clinical data on *Lactobacillus* LB suggesting relatively large effects on bowel-related outcomes [6]. Assuming an effect size of Cohen’s d *=* 0.80, a two-tailed significance level (α) of 5%, and a statistical power (1 − β) of 90%, the required sample size was estimated at 68 participants (34 per group). To maximize power (96.4%) under budgetary constraints and account for anticipated dropout rate and protocol deviation (10%), the total participants to be enrolled was set at 100 (50 per group).

Prior to unblinding, the principal investigator reviewed compliance of all subjects to decide on inclusion of the data. The Intention-To-Treat (ITT) analyses included all randomized participants who provided at least one post-baseline assessment for any endpoint. For endpoints measured only at baseline (visit 1) and at the end of study visit (visit 2), the ITT population included people who provided an end of study measurement (modified Intention-To-Treat (mITT)). No imputation was attempted leading to the exclusion of three participants.

All statistical analyses were performed using SPSS (v23). Differences were considered statistically significant at *p* < 0.05.

Efficacy was assessed using linear mixed effects models that include participants as random factors. Covariates included categorical fixed effects for group, time point, and interaction between group and time point, whereas continuous fixed effects included the baseline values and the interaction between baseline values and time points. Additionally, the analysis of covariance (ANCOVA) with baseline values as a covariate, chi-square tests, and Mann–Whitney U tests were applied as appropriate. Data was visualized using GraphPad (v10.2.2).

Safety laboratory panels were evaluated as proportion of participants whose values shifted from within the reference range at baseline visit (visit 1) to outside of the reference range at end of study visit (visit 2). Intergroup comparisons were performed using a chi-square test.

The study was powered for the primary endpoint, bowel movement frequency after four weeks of intervention. Analyses of secondary endpoints and safety laboratory parameters were considered exploratory and hypothesis-generating. Therefore, no formal adjustment for multiplicity was applied to these analyses.

## 3. Results

### 3.1. Participants’ Characteristics

Of 100 participants who started the study, 97 completed the intervention and attended the end of study visit (visit 2). Three participants discontinued the study and did not attend the end of study visit (visit 2), resulting in their classification as lost to follow-up. They provided baseline and partial post-baseline questionnaire data. The characteristics of the study’s participants are given in Table 1. The definitions of analysis populations are provided in the Material and Methods section. Both groups were well balanced for sex, age, and BMI.

### 3.2. Primary Endpoint: Improvement in Bowel Movement Frequency

**Figure 1:**
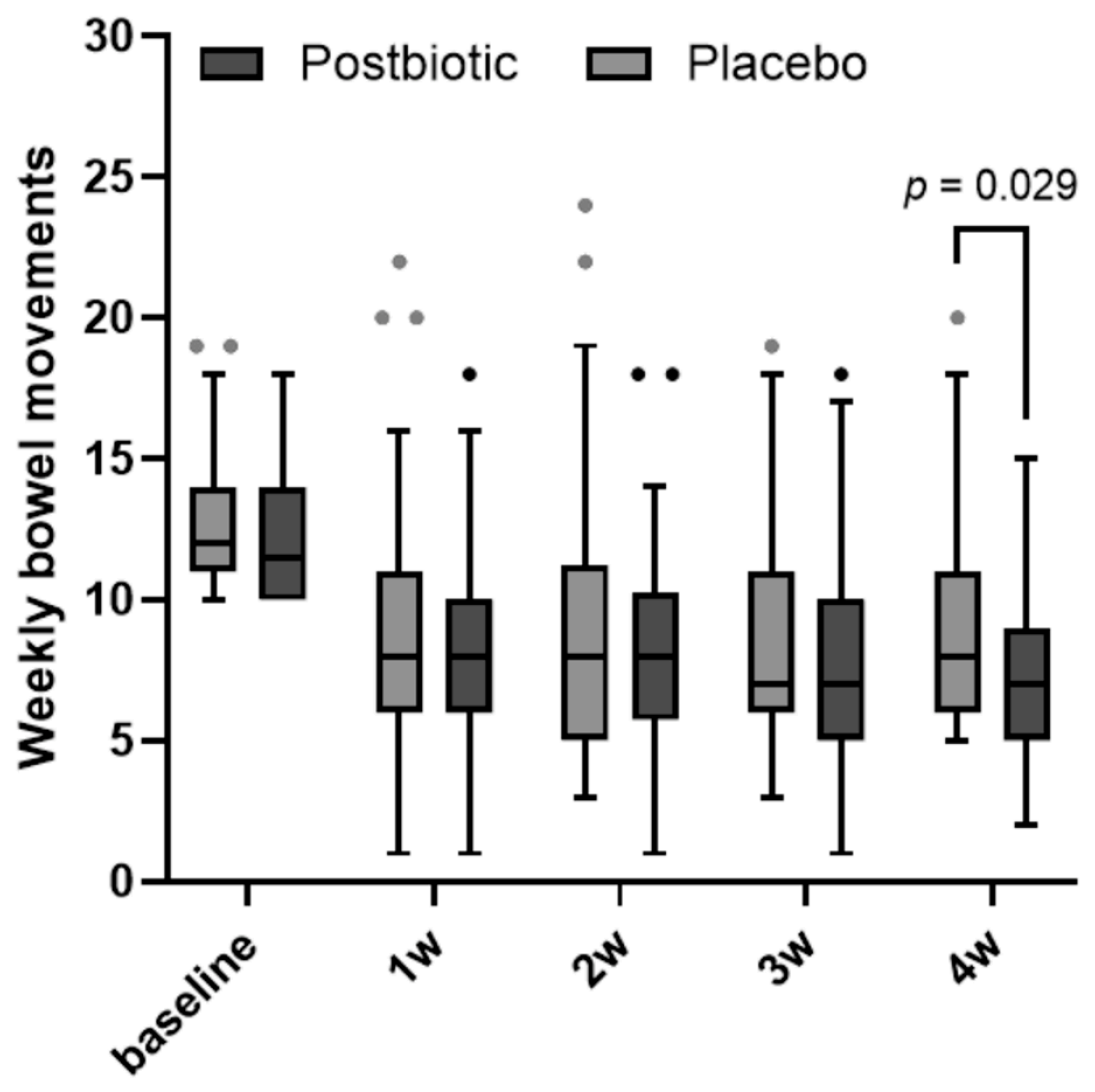
The effect of a postbiotic intervention on weekly bowel movements. Boxplots with box elements showing upper and lower quantile and median. Whiskers extend to the highest or lowest value within 1.5 times the interquartile range. Outliers are indicated as points beyond whiskers. Each timepoint represents weekly bowel movements during the week immediately preceding that timepoint. baseline: one week before intervention start; w: week of intervention.

Detailed bowel movement frequency data are provided in Table S1. At intervention start, bowel movement frequencies as recorded over the previous week (baseline) were nearly identical in both groups (postbiotic group: 12.3 ± 2.2; placebo group: 12.5 ± 2.5; Table S1). During the intervention period, weekly bowel movements gradually decreased in both groups, with no significant differences observed between groups during weeks one to three. However, after 4 weeks of intervention (4w), weekly bowel movement frequency was significantly lower in the postbiotic group compared to the placebo group (7.4 ± 3.3 versus 8.7 ± 3.5, *p =* 0.029; Figure 1).

### 3.3. Secondary Endpoints

#### 3.3.1. Days with Bowel Movements per Week, Stool Characteristics, and Sense of Relief

The average number of days with bowel movements decreased gradually over time, from 6.5 ± 0.7 and 6.6 ± 0.7 for the postbiotic and placebo group, respectively, at baseline, to 5.5 ± 1.7 and 6.3 ± 0.9 after 4 weeks of intervention. Compared to placebo, the postbiotic group had nominally significantly fewer days with bowel movements after 3 (*p* = 0.011) and 4 weeks (*p* = 0.005) of intervention (Table 2).

Bristol stool types showed no significant differences between groups throughout the study, except after 2 weeks of intervention, where type 5 (“soft blobs with clear cut edges”) was nominally significantly more common in the postbiotic group when compared with the placebo group (42% versus 20.0%; *p* = 0.032). At no time point were Bristol stool types observed consistent with severe diarrhea, i.e., type 7.

Stool color did not significantly differ between groups, except after 3 weeks of intervention, where slightly more cases of dark-brown stool color were observed in the postbiotic compared to the placebo group (8.0% versus 0.0%, *p* = 0.048), representing a nominally significant difference.

At baseline, fecal water content was nominally significantly lower in the postbiotic compared to the placebo group (76.0 ± 7.0% versus 79.1 ± 6.5%; *p* = 0.026). However, during the intervention period, the fecal water content did not significantly differ between groups.

No significant differences between groups were observed for stool volumes, stool odor, and sense of relief during bowel movements throughout the study.

#### 3.3.2. Assessment of Gastrointestinal Symptoms

Overall gastrointestinal symptoms, as assessed via the GSRS and Izumo scale, were generally mild throughout the study. Both groups showed a decrease in total GSRS and Izumo scores after a 4-week intervention compared to baseline. However, no significant differences were observed between the postbiotic and placebo groups. Detailed GSRS results are presented in Table S2 and detailed Izumo scale results in Table S3. The postbiotic group had nominally significantly lower heartburn scores at baseline compared to the placebo group (1.3 ± 0.5 versus 1.6 ± 0.8; *p* = 0.016) indicating some baseline imbalance for sub-scores.

Diarrhea sub-scores on both the GSRS and Izumo scales decreased in both groups over the intervention period. For the GSRS, diarrhea scores declined from 2.7 ± 1.4 to 2.1 ± 1.1 in the postbiotic group and from 2.6 ± 1.2 to 1.7 ± 0.7 in the placebo group. For the Izumo scale, scores decreased from 3.9 ± 3.8 to 2.8 ± 3.2 in the postbiotic group and from 3.6 ± 2.8 to 1.6 ± 1.7 in the placebo group. At week 4, diarrhea scores were nominally significantly lower in the placebo group compared to the postbiotic group (*p* < 0.05).

#### 3.3.3. Fecal Microbiome Composition

**Figure 2:**
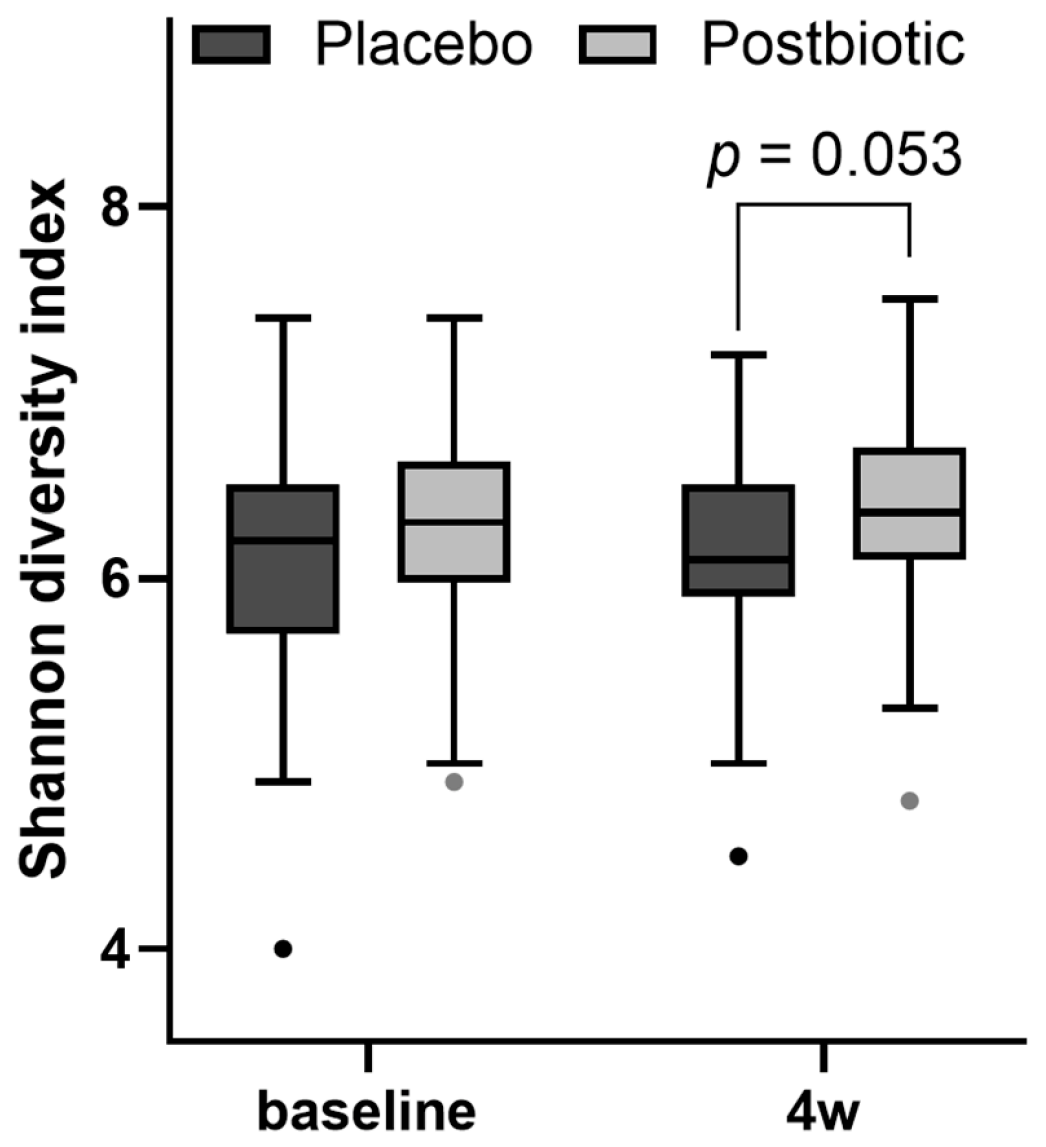
The effect of a postbiotic intervention on the Shannon diversity index. Boxplots with box elements showing upper and lower quantile and median. Whiskers extend to the highest or lowest value within 1.5 times the interquartile range. Outliers are indicated as points beyond whiskers. baseline: one week before intervention start; w: week of intervention.

To determine the effect of the postbiotic intervention on alpha diversity (diversity within a community), observed number of amplicon sequencing variants (ASVs; richness), the Faith’s phylogenetic diversity (PD) index (richness accounting for phylogenetic relationships), and the Shannon diversity index (function of evenness and richness) were calculated at baseline and after a 4-week intervention period. No significant differences in community richness (observed number of ASVs and Faith’s PD index) were observed between groups. However, there was a trend (*p* = 0.053) of increased Shannon diversity for the postbiotic group compared to the placebo (Figure 2), suggesting a redistribution of taxa abundances.

The complete taxonomic results, including relative abundances and statistical comparisons for all nominally significantly different genera, are provided in Supplementary Data File S1. At baseline, the relative abundance of ten genera showed nominally significant differences between groups (*p* < 0.05). Following the 4-week intervention period, 18 genera showed nominally significant differences (*p* < 0.05). However, effect sizes were generally small and no consistent community wide shift in microbiome composition was observed.

#### 3.3.4. Safety

No adverse events were reported for either group during the study. Nominally significant differences between groups were observed in safety laboratory blood panels for the proportion of participants whose values shifted from within the reference range at baseline visit to outside of the reference range at end of study visit. Particularly, differences in total protein (*p* = 0.026; postbiotic group: 5 cases (10.0%), placebo group: 0 cases (0.0%)), and uric acid levels (*p* = 0.026; postbiotic group: 5 cases (10.0%), placebo group: 0 cases (0.0%)) were observed. All out of range values were assessed by a qualified investigator and judged not to be clinically significant.

## 4. Discussion

Here we demonstrate that supplementation with *Lactobacillus* LB results in a modest but statistically significant reduction in bowel movement frequency and defecation days, in the absence of constipation-related effects, major microbiome changes, or safety concerns.

### 4.1. The Postbiotic Lactobacillus LB Reduced Bowel Movement Frequency in Healthy Individuals

This randomized, double-blind, placebo-controlled clinical trial evaluated the effects of a postbiotic containing heat-inactivated *Limosilactobacillus fermentum* CNCM I-2998 and *Lactobacillus delbrueckii* subsp. *lactis* CNCM I-4831, known as *Lactobacillus* LB, on bowel habits, gastrointestinal parameters and the fecal microbiome in healthy Japanese adults with self-reported frequent and soft stools. We observed a statistically significant reduction in bowel movement frequency, the primary endpoint of the study. Exploratory analyses further indicated nominally significant reductions in the number of days with bowel movements. No adverse events were reported, and nominally significant changes in laboratory blood parameters were judged not to be clinically significant by the qualified investigator. Although the participants included in this study did not meet the diagnostic criteria for functional diarrhea or diarrhea-predominant IBS as defined by Rome IV criteria [13], they were selected specifically because they reported unusually frequent soft stools. Recent population-based research suggests that bowel movement frequency is associated with distinct microbial and metabolomic profiles even among generally healthy adults, suggesting that bowel habits may be a relevant marker of gastrointestinal and systemic physiology beyond clinically diagnosed gastrointestinal disorders [2]. Accordingly, this population is representative of individuals who experience bowel habits outside their preferred range and who may seek support for bowel habit regulation despite the absence of a clinically diagnosed gastrointestinal disorder. Individuals with higher-than-desired bowel movement frequency may be encountered in clinical practice and population-based studies. In line with our findings, another study showed that administration of probiotic fermented milk for 4 weeks significantly decreased defecation frequency compared with baseline [14], suggesting that modulation of bowel movement frequency is a shared functional outcome across microbiome-targeting interventions, including both probiotics and postbiotics — thus, further supporting the notion that non-viable microbial preparations can be effective in modulating gastrointestinal function by exerting mechanisms of action that may be shared with probiotics [15].

The reduction of approximately 1.3 bowel movements per week observed versus placebo in this study should be interpreted within the context of the study population. Epidemiological data indicate that stool frequency in healthy adults may comprise a wide range, but sustained increases beyond one to two movements a day may be associated with functional symptoms, impairing quality of life [16]. Rather than inducing large changes in bowel movement frequency typical for pharmacological antidiarrheal treatments, the observed effects were characterized by a modest decrease in bowel movement frequency and number of defecation days. It is also interesting to note the absence of a compensatory increase in constipation-related endpoints. Bowel movement frequency in the postbiotic group remained within satisfactory ranges for healthy adults [16], and no decrease in stool volume, increase in hard stools or constipation symptoms were reported. This distinction is important, as an excessive suppression of bowel movements is a known limitation of antidiarrheal drugs [17], whereas healthy individuals may seek support for maintaining regularity rather than interventions that risk inducing constipation. Exploratory analyses indicated the simultaneous reduction in the number of defecation days together with stable stool volumes suggesting that the postbiotic may have changed bowel rhythm rather than just stool output. However, the nature and extent of such changes were not directly assessed and should be examined in future studies.

Exploratory changes in stool form were modest and temporally variable. A transient increase in Bristol stool type 5 during the mid-intervention period was observed in the postbiotic group, while no sustained shift toward firmer stool forms was detected at the end of the intervention. Although stool frequency and stool consistency are related measures of bowel function, they do not always change in parallel. This has been reported previously in gastroenterology research and likely reflects the multifactorial determinants of stool form, including intestinal transit time, fermentation activity, bile acid metabolism, and colonic water handling [10]. Together, these findings suggest that stool frequency and stool consistency represent related but distinct dimensions of bowel habits, each providing complementary insights into gastrointestinal function.

Despite changes in bowel movement frequency, fecal water content did not differ significantly between groups at the end of the intervention. This finding suggests that the observed effects were not driven primarily by alterations in luminal water retention or secretory processes. Nevertheless, stool frequency and fecal water content do not necessarily correlate linearly, as observed in previous studies, particularly in individuals with functional bowel disturbances or subclinical diarrhea [18].

A sustained reduction in bowel movement frequency was observed in the placebo group as well. Placebo responses are well documented in gastrointestinal trials, particularly in conditions involving bowel habits and symptom perception. For example, Kaptchuk et al. (2008) [19] demonstrated significant improvements in bowel symptoms among placebo-treated patients with IBS, highlighting the influence of expectation and the brain–gut axis. Similarly, Enck et al. (2012) [20] reported placebo effects on stool frequency and gastrointestinal symptom scores across multiple IBS trials. The magnitude of improvement observed in the placebo group highlights the importance of placebo-controlled study designs when evaluating bowel habit-related outcomes. Nevertheless, despite this substantial placebo response, a statistically significant difference between groups remained evident after four weeks of intervention. Accordingly, the observed between-group difference should be interpreted as a modest treatment effect beyond the background placebo response.

### 4.2. Potential Mechanisms of Action of Lactobacillus LB

Several mechanisms that may influence gut motility have been proposed in the literature, including interactions between epithelial, neuronal, immune, and gut microbial pathways [21,22]. Modulation of such pathways via postbiotics is biologically plausible as they may contain a range of bioactive microbial structures that can interact with host tissues. Heat-inactivated lactobacilli retain structural cell components such as peptidoglycans, lipoteichoic acids, surface layer proteins, and bacterial DNA fragments. These bioactives can interact with intestinal epithelial cells and immune cell receptors, including toll-like receptors and nucleotide-binding oligomerization domain receptors. These microbial-associated molecular patterns may promote epithelial barrier integrity, mucosal immunity, and enteric nervous system signaling [4]. Consistent with these hypotheses, previous in vitro studies investigating the presently studied postbiotic have reported effects on epithelial barrier integrity, immunity, and microbial composition. Mouse studies using the presently studied postbiotic demonstrated that inactivated lactobacilli significantly influence ileal ion transport ex vivo and had an inhibitory effect on intestinal tone and increased cholinergic-induced contractility [23]. In addition, in an organoid model, it has been shown that *Lactobacillus* LB mitigates drug-induced epithelial damage, promotes epithelial recovery, and shows anti-inflammatory effects [24]. Similarly, in a mouse model of colitis, this postbiotic led to a reduction in intestinal inflammation as assessed via small intestine and colon crypt length [25]. Collectively, these observations provide plausible mechanistic hypotheses through which the postbiotic may influence gastrointestinal motility.

A bifidogenic effect in pure culture and in human fermented fecal communities was observed upon addition of *Lactobacillus* LB [8], suggesting that modulation of microbial community may contribute to the biological activity of the postbiotic. Although only mild between-group differences in overall gut microbiome composition were observed after four weeks of intervention in the present study, this finding is consistent with previous studies using biotics indicating that functional gastrointestinal effects may occur without detectable taxonomic shifts [26]. The absence of large-scale microbiome compositional changes does not preclude functional modulation. Increasing evidence suggests that microbial metabolic output and host–microbe signaling may be more relevant to gastrointestinal function than taxonomic shifts alone, particularly in short-term interventions [27]. The present findings are consistent with the hypothesis that *Lactobacillus* LB may influence gastrointestinal motility through effects on microbial metabolic activity or host–microbe signaling rather than large changes in microbial abundance. However, the present clinical study was not designed to evaluate these mechanisms directly, and therefore no conclusions regarding the underlying mode of action can be drawn from the current data.

### 4.3. Patient-Reported Results and Symptom Perception

Self-reported outcomes assessed using the GSRS and the Izumo Scale provided mixed results. While objective bowel movement frequency measures were favored for the postbiotic group, exploratory diarrhea-related symptom scores were higher at the end of the intervention for this group when compared to the placebo. This apparent discrepancy highlights the inherent complexity of the perception and report of symptoms by healthy people with a low baseline of the burden of symptoms. Symptom perception is a multifactorial phenomenon that includes visceral sensitivity, cognitive appraisal, attention to bodily sensations and the overall psychological context [28]. One possible explanation is that the increased awareness of bowel habits during trial participation may paradoxically amplify symptom reporting, even in the absence of objective worsening, although this hypothesis was not directly assessed in the present study. In addition, we must consider that symptom scales such as the GSRS were developed primarily for symptomatic populations and that they may have limited sensitivity or specificity in healthy populations. It is worth noting that no deterioration in overall GSRS or Izumo total scores was observed, nor an increase in the number of adverse gastrointestinal events. These findings suggest that the postbiotic intervention was well tolerated and did not negatively impact overall gastrointestinal well-being. The divergence between objective and subjective outcomes should therefore be interpreted cautiously and should not undermine the relevance of the findings related to bowel frequency.

The present findings are consistent with previous clinical studies investigating the same postbiotic product but in individuals with different gastrointestinal conditions. In a one-arm observational investigation in 297 patients with diarrhea-predominant IBS, *Lactobacillus* LB was effective in reducing the number of stools per week, abdominal pain and bloating [29]. In a randomized, double-blind, cross-over trial, the same postbiotic led to a statistically significant therapeutic benefit in 50% of patients, when all of the six selected clinical criteria representative of IBS were considered [30]. In people with chronic diarrhea, *Lactobacillus* LB given for four weeks proved to be more effective than living lactobacilli in the treatment of this condition [6]. The current study extends these observations to a healthy Japanese population with subclinical increased frequent and soft stools. This is an important distinction, as previous studies focused on symptomatic patients.

### 4.4. Strengths and Limitations

This study has several strengths including its randomized, double-blind, placebo-controlled design, the use of a statistically estimated sample size, high participant compliance, and the longitudinal assessment of bowel habits using validated tools. However, some limitations should be acknowledged.

Several baseline differences were observed despite randomization, including fecal water content, heartburn scores, and the relative abundance of microbial genera. Although baseline values were incorporated into the statistical models where applicable, residual effects of the baseline imbalances cannot be completely excluded. Furthermore, while the present study was designed and powered to evaluate bowel movement frequency at the end of the intervention as the primary endpoint, numerous secondary outcomes were also assessed. No adjustment for multiplicity was applied across these analyses, as they were treated as exploratory outcomes. Consequently, findings related to secondary outcomes should be interpreted cautiously as exploratory observations and warrant confirmation in future studies.

The intervention was conducted for four weeks. While appropriate for assessing changes in stool frequency, longer-term studies are warranted to further explore additional benefits, such as more robust microbiome modulations. In addition, the present study was not designed or powered to evaluate potential differences in response according to demographic or dietary characteristics. Future studies with larger sample sizes may help determine whether factors such as age, sex, dietary habits, or baseline gut microbiome composition influence the responsiveness to the postbiotic.

The study population consisted of healthy Japanese adults; although dietary patterns may influence gut microbiome composition and thus limit generalizability, only mild changes in the gut microbiome composition were observed. This suggests that the mechanism of action may be at least partially microbiome-independent and therefore likely to be generalized to other populations. Additionally, expanding endpoints to include biomarkers such as short chain fatty acids, bile acids or zonulin, among others, might provide additional insights into the underlying mechanisms of actions of the effects observed.

## 5. Conclusions

In this study, we demonstrated that the postbiotic containing heat-inactivated *Limosilactobacillus fermentum* and *Lactobacillus delbrueckii* subsp. *lactis* modestly but significantly reduced bowel movement frequency in healthy Japanese adults with self-reported frequent and soft stools, without raising safety concerns. A reduction in the exploratory endpoint of defecation days was also observed. However, no mechanistic endpoints were included in this study to provide direct insight into the biological processes underlying the observed effects. Future studies incorporating longer intervention periods, mechanistic endpoints, and possibly analyses considering responders and non-responders would enable a more comprehensive understanding of the observed effects. Such research will provide further evidence on the role of postbiotics to support gut health in non-diseased populations.

## Figures and Tables

**Table 1:** Study participants’ characteristics.

Parameter		ITT		mITT	
		Postbiotic	Placebo	Postbiotic	Placebo
Sex	Male (n (%))	25 (50.0%)	25 (50.0%)	25 (50.0%)	23 (48.9%)
	Female (n (%))	25 (50.0%)	25 (50.0%)	25 (50.0%)	24 (51.1%)
Age (years)	Mean (SD)	44.8 (13.6)	42.7 (11.7)	44.8 (13.6)	42.5 (12.0)
BMI (kg/m^2^)	Mean (SD)	22.5 (3.0)	22.4 (3.1)	22.5 (3.0)	22.5 (3.2)

BMI: body mass index; ITT: Intention-To-Treat; mITT: modified Intention-To-Treat; SD: standard deviation.

**Table 2:** The effect of a postbiotic intervention on days with bowel movements per week. Statistical significance was assessed using Welch’s t-test at baseline and a linear mixed model during weeks one to four of the intervention (1w–4w).

Study Week	Postbiotic				Placebo				Group Comparison				
	Mean (SD)	EMM (SE)	95%CI−	95%CI+	Mean (SD)	EMM (SE)	95%CI−	95%CI+	∆	SE	95%CI−	95%CI+	*p*
Baseline	6.5 (0.7)	-	-	-	6.6 (0.7)	-	-	-	−0.1	0.1	−0.3	0.2	0.665
1w	5.7 (1.6)	5.7 (0.2)	5.3	6.1	6.1 (1.4)	6.0 (0.2)	5.6	6.4	−0.3	0.3	−0.9	0.2	0.263
2w	5.5 (1.7)	5.5 (0.2)	5.1	5.9	5.9 (1.3)	5.9 (0.2)	5.5	6.3	−0.4	0.3	−1.0	0.2	0.172
3w	5.5 (1.6)	5.5 (0.2)	5.1	5.9	6.3 (1.2)	6.2 (0.2)	5.8	6.6	−0.7	0.3	−1.3	−0.2	0.011
4w	5.5 (1.7)	5.5 (0.2)	5.1	5.9	6.3 (0.9)	6.2 (0.2)	5.9	6.6	−0.8	0.3	−1.3	−0.2	0.005

Baseline: one week before intervention start; EMM: Estimated marginal mean; *p*: statistical significance; SE: Standard error; SD: standard deviation; w: week of intervention; ∆: Intergroup difference (postbiotic—placebo group); 95%CI−: 95% confidence interval lower limit; 95%CI+: 95% confidence interval upper limit.

## Data Availability

The study protocol is available at https://center6.umin.ac.jp/cgi-open-bin/ctr_e/ctr_view.cgi?recptno=R000058715 (accessed on 27 January 2026). The individual data is not publicly available due to privacy legislation. Requests will be evaluated regarding the legitimacy of the research question and compatibility with privacy legislation.

## Supplementary Materials

The following supporting information can be downloaded at: https://www.mdpi.com/article/10.3390/microorganisms14091939/s1, Table S1: The effect of a postbiotic intervention on bowel movement frequency; Table S2: The effect of a postbiotic intervention on overall GSRS scores; Table S3: The effect of a postbiotic intervention on overall Izumo scores; Supplementary Data File S1: The effect of a postbiotic intervention on fecal microbiome composition.

## Abbreviations

The following abbreviations are used in this manuscript:

ASVs	Amplicon sequencing variants
ANCOVA	Analysis of covariance
GSRS	Gastrointestinal Symptoms Rating Scale
IBS	Irritable Bowel Syndrome
ITT	Intention-To-Treat
mITT	modified Intention-To-Treat
