# Supplementary material for: A Postbiotic Containing Limosilactobacillus fermentum CNCM I-2998 and Lactobacillus delbrueckii subsp. lactis CNCM I-4831 Reduces Bowel Movement Frequency in Healthy Adults with Self-Reported Unusually Frequent and Soft Stools
Source: Microorganisms. 2026 Sep 2;14(9):1939. doi: 10.3390/microorganisms14091939 (PMC13609219; doi:10.3390/microorganisms14091939)
Supplement: Supplementary file 1 [file microorganisms-14-01939-s001.zip › Supplementary-Material.pdf]

Supplementary Material:

Table S1: The effect of a postbiotic intervention on bowel movement frequency. Statistical significance was assessed using Welch's t-test at baseline and a linear mixed effect model during weeks one to four of the intervention (1w-4w).

Study week	Postbiotic				Placebo				Group comparison				
	Mean (SD)	EMM (SE)	95%CI−	95%CI+	Mean (SD)	EMM (SE)	95%CI−	95%CI+	Δ	SE	95%CI−	95%CI+	*p*
Baseline	12.3 (2.2)	-	-	-	12.5 (2.5)	-	-	-	−0.2	0.5	−1.2	0.7	0.611
1w	8.1 (3.6)	8.2 (0.2)	7.4	9.0	9.0 (4.4)	8.9 (0.4)	8.0	9.7	−0.7	0.6	−1.9	0.5	0.269
2w	8.0 (3.7)	8.1 (0.2)	7.2	9.1	9.0 (4.8)	8.9 (0.5)	7.9	9.9	−0.8	0.7	−2.1	0.6	0.278
3w	7.7 (3.7)	7.8 (0.2)	7.0	8.7	8.5 (3.8)	8.5 (0.4)	7.7	9.4	−0.7	0.6	−1.9	0.5	0.255
4w	7.4 (3.3)	7.4 (0.2)	6.6	8.2	8.7 (3.5)	8.7 (0.4)	7.9	9.5	−1.3	0.6	−2.4	−0.1	0.029

Baseline: one week before intervention start; w: week of intervention; EMM: Estimated marginal mean; SE: Standard error; SD: standard deviation; Δ **:** Intergroup difference (postbiotic − placebo group); 95%CI−: 95% confidence interval lower limit; 95%CI+: 95% confidence interval upper limit; *p*: statistical significance

Table S2: The effect of a postbiotic intervention on overall GSRS scores. Statistical significance was assessed using Welch's t test at baseline and an ANCOVA at 4 weeks (4w), with baseline as a covariate and group (postbiotic, placebo) as factors.

Study week	Postbiotic				Placebo				Group comparison				
	Mean (SD)	EMM (SE)	95%CI−	95%CI+	Mean (SD)	EMM (SE)	95%CI−	95%CI+	Δ	SE	95%CI−	95%CI+	*p*
**Baseline**	1.9 (0.7)	-	-	-	2.0 (0.6)	-	-	-	−0.1	0.1	−0.4	0.2	0.459
**4w**	1.8 (0.7)	1.8 (0.1)	1.6	1.9	1.7 (0.5)	1.7 (0.1)	1.5	1.8	0.1	0.1	−0.1	0.3	0.316

Baseline: one week before intervention start; w: week of intervention; EMM: Estimated marginal mean; SE: Standard error; SD: standard deviation; Δ**:** Intergroup difference (postbiotic − placebo group); 95%CI−: 95% confidence interval lower limit; 95%CI+: 95% confidence interval upper limit; *p*: statistical significance

Table S3: The effect of a postbiotic intervention on overall Izumo scores. Statistical significance was assessed using Welch's t test at baseline and an ANCOVA at 4 weeks (4w), with baseline as a covariate and group (postbiotic, placebo) as factors.

Study week	Postbiotic				Placebo				Group comparison				
	Mean (SD)	EMM (SE)	95%CI−	95%CI+	Mean (SD)	EMM (SE)	95%CI−	95%CI+	Δ	SE	95%CI−	95%CI+	*p*
**Baseline**	10.8 (8.7)	-	-	-	10.7 (9.2)	-	-	-	0.1	1.8	−3.5	3.7	0.948
**4w**	8.6 (8.2)	8.5 (0.9)	6.7	10.4	6.8 (6.1)	6.8 (0.9)	4.9	8.7	1.7	1.3	−0.9	4.4	0.187

Baseline: one week before intervention start; w: week of intervention; EMM: Estimated marginal mean; SE: Standard error; SD: standard deviation; Δ**:** Intergroup difference (postbiotic − placebo group); 95%CI−: 95% confidence interval lower limit; 95%CI+: 95% confidence interval upper limit; *p*: Significance probability
